# CNIH4 as a prognostic biomarker and promoter of malignant progression in head and neck squamous cell carcinoma

**DOI:** 10.3389/fonc.2026.1963295

**Published:** 2026-10-13

**Authors:** Jun Chen, Aitao Nai, Jijie Cai, Mingchu Liao, Suli Lu, Junyan He

**Affiliations:** 1 Department of Oncology, The First Affiliated Hospital, Hengyang Medical School, University of South China, Hengyang, China; 2 Department of Anatomy, Medical College of Hunan Polytechnic of Environment and Biology, Hengyang, China

**Keywords:** CNIH4, head and neck squamous cell carcinoma, oral squamous cell carcinoma, prognostic biomarker, RNA sequencing, tumor immune microenvironment

## Abstract

**Background:**

Head and neck squamous cell carcinoma (HNSCC) is a molecularly heterogeneous malignancy with variable clinical outcomes. Although cornichon family AMPA receptor auxiliary protein 4 (CNIH4) has been implicated in several disease processes, its clinical and biological relevance in HNSCC remains incompletely defined.

**Methods:**

Transcriptomic, genomic, and clinical data from The Cancer Genome Atlas and independent Gene Expression Omnibus cohorts were integrated for bioinformatic analyses. CNIH4 expression was evaluated in relation to diagnostic performance, clinicopathological features, survival, somatic mutations, tumor mutation burden, immune infiltration, immune-regulatory genes, and drug-response profiles. A prognostic nomogram incorporating CNIH4 was also assessed. Functional studies were performed in SAS and SCC-9 oral squamous cell carcinoma cells using two independent CNIH4-targeting siRNAs. Proliferation, colony formation, and wound closure were measured, followed by RNA sequencing and pathway analyses after CNIH4 knockdown.

**Results:**

CNIH4 was consistently overexpressed in HNSCC and validated in three independent cohorts, with retrospective discrimination between tumor and normal tissues in the TCGA-HNSC transcriptomic dataset (AUC = 0.915). Higher CNIH4 expression was associated with unfavorable clinicopathological features and shorter overall survival, disease-specific survival, disease-free interval, and progression-free interval. The nomogram showed moderate prognostic performance, with AUCs of 0.710, 0.750, and 0.720 at 1, 3, and 5 years, respectively. CNIH4 expression was also associated with distinct mutation patterns, higher tumor mutation burden, and differences in bulk transcriptome-derived immune and stromal signatures and immune-regulatory gene expression. Higher CNIH4 expression correlated with lower predicted IC50 values for fluorouracil, tegafur, paclitaxel, and nilotinib. Experimentally, CNIH4 depletion reduced proliferation, colony formation, and wound closure in both cell lines. RNA sequencing revealed changes involving epithelial–mesenchymal transition, interferon signaling, TNF-α/NF-κB signaling, NOTCH signaling, p53-related programs, and the unfolded protein response.

**Conclusions:**

This study suggests that CNIH4 is associated with HNSCC progression-related phenotypes and clinical outcomes. Functional and transcriptomic findings support a functional association between CNIH4 and malignant phenotypes in oral squamous cell carcinoma and justify further investigation of its prognostic and therapeutic relevance in HNSCC.

## 1 Introduction

Head and neck squamous cell carcinoma (HNSCC) encompasses malignancies arising from the mucosal surfaces of the oral cavity, pharynx, and larynx. Tobacco and alcohol exposure remain major etiological factors, while infection with high-risk human papillomavirus defines a biologically and clinically distinct subset of disease (1, 2). Current management combines surgery, radiotherapy, systemic chemotherapy, targeted agents, and immune checkpoint blockade according to tumor site and stage. Nevertheless, recurrence, distant dissemination, and treatment resistance continue to limit long-term disease control. In addition, both tumor progression and intensive multimodal treatment can substantially impair swallowing, speech, breathing, and quality of life. Improved molecular markers are therefore needed to refine disease characterization, outcome estimation, and therapeutic stratification.

The clinical diversity of HNSCC reflects substantial heterogeneity at both the genomic and microenvironmental levels. Large-scale molecular profiling has identified recurrent alterations involving TP53, CDKN2A, PIK3CA, and cell-cycle regulatory pathways, with important differences between HPV-associated and HPV-negative tumors (2). At the same time, malignant cells interact with heterogeneous lymphoid, myeloid, and stromal populations that can modify tumor growth and treatment response. Immune-regulatory molecules and the abundance or functional state of infiltrating immune cells also vary considerably among tumors. Although HPV status and programmed death-ligand 1 expression provide clinically useful information in selected settings, neither fully captures this biological diversity. Molecular features that integrate tumor-intrinsic behavior with clinical and immune characteristics may therefore provide additional insight into HNSCC progression.

Cornichon family member 4 (CNIH4) is a transmembrane protein involved in the intracellular handling of newly synthesized proteins. It has been reported to interact with the coat protein complex II machinery and to facilitate endoplasmic reticulum export of selected G-protein-coupled receptors (3). Through effects on membrane-protein trafficking, secretory processes, and proteostasis, CNIH4 may influence tumor-cell signaling and adaptation to microenvironmental stress. These processes could also be relevant to tumor–microenvironment interactions; however, a direct role of CNIH4 in immune-cell recruitment, activation, or immunotherapy response has not been established. In colon cancer, CNIH4 participates in a TMED9–TGF-α–GLI signaling network, and its depletion was associated with reduced migratory and metastatic properties (4). In HNSCC, a previous database-based analysis reported increased CNIH4 expression and associations with patient survival, cancer stemness, immune infiltration, and predicted response to immune checkpoint blockade (5). These findings provided an important hypothesis-generating framework, but were derived predominantly from computational analyses and did not determine whether CNIH4 functionally contributes to malignant phenotypes in HNSCC cells or identify transcriptomic changes following its depletion.

In the present study, we first sought to validate and extend the previously reported expression and clinical associations of CNIH4 using TCGA-HNSC and independent GEO cohorts. These analyses, including associations with clinicopathological features, survival, somatic mutation patterns, tumor mutation burden, immune-related signatures, and drug-response datasets, should primarily be interpreted as confirmatory or exploratory extensions of prior computational observations. The principal novel component of this study was the functional evaluation of CNIH4 in oral squamous cell carcinoma cells. CNIH4 was depleted using two independent siRNAs in SAS and SCC-9 cells to assess effects on proliferation, clonogenic growth, and wound-closure capacity. RNA sequencing was subsequently performed after CNIH4 knockdown to identify candidate transcriptional programs associated with the observed phenotypes. By integrating multi-cohort analyses with functional experiments and transcriptomic profiling, we aimed to move beyond correlative database observations and provide evidence that CNIH4 is functionally associated with malignant behavior in oral squamous cell carcinoma, while keeping its immune and therapeutic implications appropriately hypothesis-generating.

## 2 Materials and methods

### 2.1 Data acquisition and preprocessing

The TCGA Head and Neck Squamous Cell Carcinoma project (TCGA-HNSC) was used as the principal cohort for transcriptomic, clinicopathological, survival, and somatic mutation analyses. Transcriptomic profiles from 33 TCGA cancer types were additionally included for pan-cancer comparisons. Mutation annotation format files and the corresponding clinical outcome records were retrieved for analyses of genomic alterations and patient survival, respectively (2).

Three GEO cohorts, GSE23558, GSE78060, and GSE30784, were independently analyzed to assess whether the differential expression of CNIH4 observed in TCGA-HNSC could be reproduced in external datasets (6). GSE23558 is an oral squamous cell carcinoma (OSCC) microarray cohort comprising 27 tumor samples and 5 normal oral tissue samples. GSE78060 is a microarray cohort of advanced tongue squamous cell carcinoma (TSCC), in which 26 tumor samples and 4 normal tissue samples were included in the present tumor-versus-normal analysis. GSE30784 is an OSCC microarray cohort comprising 167 tumor samples and 45 normal oral tissue samples. These cohorts were analyzed separately because of differences in tissue composition, cohort characteristics, and microarray platforms. The association between CNIH4 expression and outcomes after immune checkpoint blockade was further examined in the IMvigor210 and GSE91061 immunotherapy cohorts.

For microarray-based cohorts, normalized expression matrices and platform annotation files were obtained from GEO. Probe identifiers were converted to official gene symbols according to the corresponding platform annotations. When more than one probe mapped to the same gene, the median probe intensity was used to represent gene-level expression. Log2 transformation was applied when required by the distribution of the original expression values.

Analyses were conducted independently within each cohort, without directly combining data generated on different platforms. Samples missing variables required for a given analysis were omitted only from that specific analysis. TCGA and GEO datasets were publicly available and de-identified; therefore, no additional ethical approval or informed consent was required for their secondary analysis. All public transcriptomic datasets were analyzed at the gene level using the official gene symbol CNIH4. Therefore, the TCGA, GEO, NCI-60, and RNA-sequencing analyses reflected aggregate CNIH4 gene expression and did not distinguish individual alternatively spliced transcript variants.

### 2.2 Pan-cancer and HNSCC expression analyses

CNIH4 expression was first surveyed across TCGA cancer types using R, with TCGAplot and ggplot2 used for data visualization. Tumor and normal tissues were compared within each available cancer type. Where matched adjacent normal tissues were available, paired analyses were additionally performed to account for interindividual variation.

Expression analysis was then focused on TCGA-HNSC. CNIH4 levels were compared between independent tumor and normal samples as well as between paired tumor and adjacent normal specimens. The same expression pattern was subsequently examined in GSE23558, GSE78060, and GSE30784. The Wilcoxon rank-sum test was used for comparisons between independent groups, whereas matched tumor-normal pairs were analyzed with the Wilcoxon signed-rank test.

### 2.3 Diagnostic and clinicopathological association analyses

The ability of CNIH4 expression to discriminate HNSCC from normal tissue was quantified by receiver operating characteristic analysis using the pROC package in R (7). Diagnostic performance was summarized by the area under the curve together with its 95% confidence interval.

Clinicopathological associations were examined within TCGA-HNSC. Variables included age, sex, primary therapy outcome, vital status, clinical T/N/M categories, overall clinical stage, pathological T/N/M categories, and overall pathological stage. Age was analyzed after classification into ≤60-year and >60-year groups. Primary therapy status was categorized as sensitive or resistant according to the available clinical response information.

For clinicopathological variables consisting of two categories, differences in CNIH4 expression were tested with the Wilcoxon rank-sum test. Variables containing more than two levels were first evaluated using the Kruskal–Wallis test, with pairwise Wilcoxon testing performed where appropriate.

### 2.4 Prognostic analyses

The prognostic relevance of CNIH4 was first screened across individual TCGA tumor cohorts. A separate univariate Cox model was fitted for each cancer type, and the resulting effect estimates were reported as hazard ratios with 95% confidence intervals.

For each outcome, surv_cutpoint() from the survminer package was used to identify the CNIH4 expression value that maximized survival separation within the TCGA-HNSC cohort. The resulting cutoff defined the CNIH4-high and CNIH4-low strata used for Kaplan–Meier visualization of overall survival, disease-specific survival, disease-free interval, and progression-free interval. Because these cutoffs were selected using outcome information from the same cohort, the dichotomized survival analyses were considered exploratory and were not interpreted as clinically validated threshold-based prognostic models.

Time-to-event distributions were estimated for both strata and displayed graphically using the Kaplan–Meier method, with statistical evidence for between-group separation obtained from a log-rank comparison. An analogous analysis was subsequently carried out in IMvigor210 and GSE91061 to examine CNIH4 expression in patients exposed to immune checkpoint blockade.

Because the cutoff generated by surv_cutpoint() was selected using survival information within the TCGA-HNSC cohort, the resulting dichotomized survival analyses were considered exploratory and were used primarily for visualization of survival separation. The prognostic relevance of CNIH4 should therefore be interpreted cautiously pending validation in an independent HNSCC cohort.

### 2.5 Genomic alteration and tumor mutation burden analyses

Pan-cancer information on CNIH4 genomic events was obtained from the Cancer Type Summary view in cBioPortal (8, 9), covering mutations, structural variants, copy-number gains and losses, and cases containing multiple alteration types.

Somatic variant data from TCGA-HNSC were analyzed in R with maftools (10). For the somatic mutation and TMB analyses, TCGA-HNSC samples were dichotomized at the cohort-specific median CNIH4 expression value, yielding CNIH4-low and CNIH4-high groups of 248 cases each. Recurrently altered genes and mutation classes were summarized with oncoplots.

For each gene, the numbers of mutated and nonmutated tumors in the two CNIH4 strata were compared by Fisher’s exact test. Odds ratios and their 95% confidence intervals were used to summarize the relative enrichment of mutations between groups.

TMB was calculated from eligible nonsynonymous somatic variants relative to the coding-region size and was log10 transformed before visualization. Its distribution was compared between the two CNIH4 strata. CNIH4 status was then combined with TMB status to generate four patient categories, whose overall survival distributions were compared by log-rank analysis.

### 2.6 Construction and evaluation of the prognostic nomogram

CNIH4 expression was incorporated with age, clinical T, N and M categories, overall clinical stage, lymphovascular invasion, and perineural invasion to construct a Cox-based survival model. Only cases with complete information for all model variables were retained.

The fitted model was represented as a nomogram with the rms package, from which 1-, 3-, and 5-year overall survival estimates were obtained. Agreement between model-derived probabilities and observed outcomes was assessed using calibration curves. Changes in discrimination over follow-up were quantified with the time-dependent C-index calculated by riskRegression and compared with the risk-score model displayed in **Figure 1c**.

**Figure 1:**
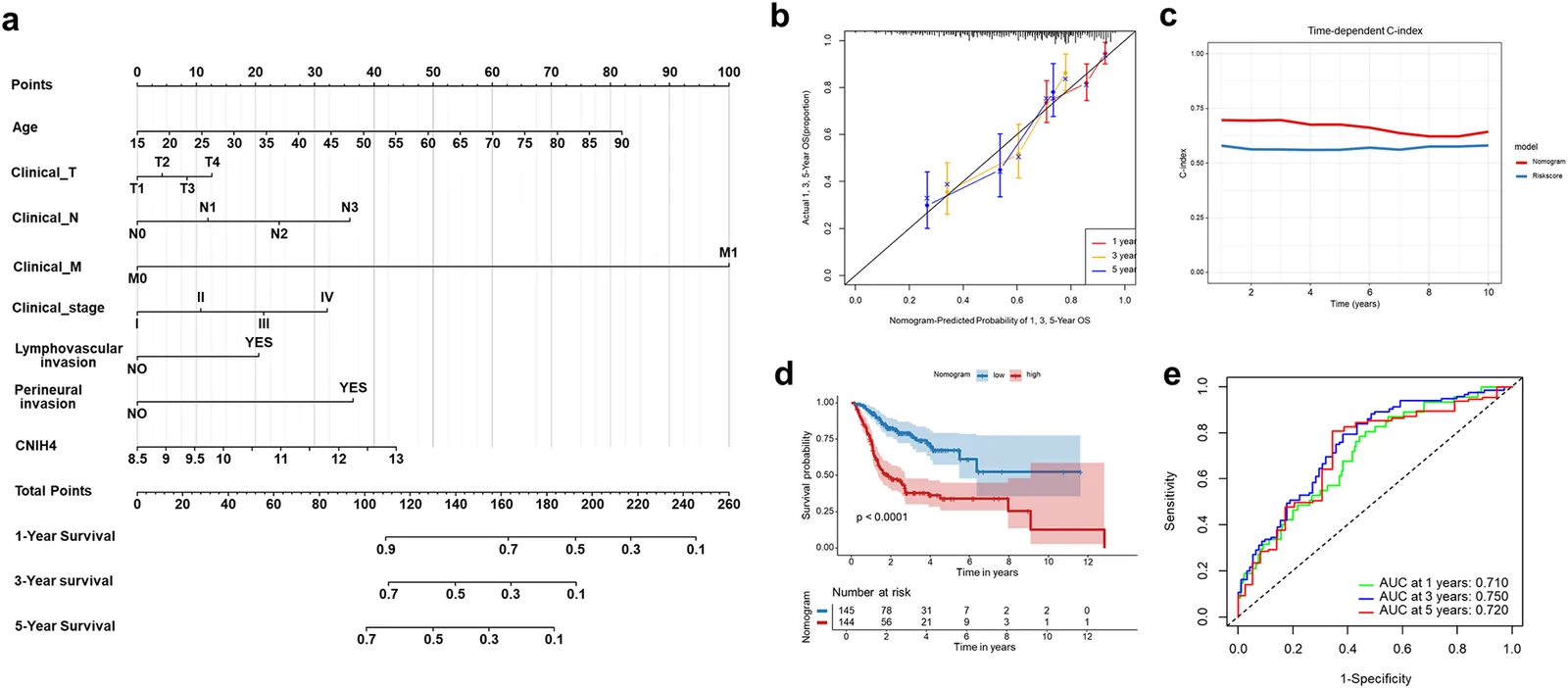
Construction and evaluation of a nomogram incorporating CNIH4 expression and clinicopathological characteristics for predicting overall survival in patients with HNSC. **(A)** A nomogram integrating age, clinical T stage, clinical N stage, clinical M stage, overall clinical stage, lymphovascular invasion, perineural invasion, and CNIH4 expression was constructed to predict the 1-, 3-, and 5-year overall survival probabilities of patients with HNSC. The points assigned to each variable were summed to obtain a total score, which was subsequently used to estimate survival probability. **(B)** Calibration curves for the nomogram-predicted 1-, 3-, and 5-year overall survival. The x-axis represents the predicted survival probability, and the y-axis represents the observed survival probability. The 45° diagonal line indicates ideal agreement. **(C)** Comparison of the time-dependent concordance index (C-index) between the nomogram and the risk-score model. **(D)** Kaplan–Meier overall survival curves for patients stratified into high- and low-nomogram-score groups. Differences between the groups were evaluated using the log-rank test. **(E)** Time-dependent receiver operating characteristic curves evaluating the ability of the nomogram to predict 1-, 3-, and 5-year overall survival, with corresponding AUC values of 0.710, 0.750, and 0.720, respectively.

The total nomogram score was also used for patient stratification. Survival between the two score strata was compared using Kaplan–Meier analysis, and prediction at 1, 3, and 5 years was evaluated with time-dependent ROC curves generated by timeROC (11).

### 2.7 Tumor microenvironment and immune infiltration analyses

For group-based ESTIMATE and ssGSEA analyses, TCGA-HNSC samples were dichotomized according to the cohort-specific median CNIH4 expression value. ESTIMATE was applied to TCGA-HNSC expression profiles to derive stromal, immune, and combined ESTIMATE scores for individual tumors (12). Score distributions were subsequently contrasted according to CNIH4 expression status.

A second analysis quantified 28 immune-cell signatures using ssGSEA implemented in GSVA (13). Sample-level patterns were visualized in a heatmap, and distributions of individual immune-cell scores were examined between CNIH4 expression groups.

CNIH4 was additionally analyzed on its original continuous scale. For each immune-cell signature, Pearson’s R was calculated against CNIH4 expression, and representative relationships were visualized with fitted trends and 95% confidence bands (14). Because ESTIMATE and ssGSEA are bulk transcriptome-based computational approaches, the resulting scores were interpreted as relative immune- and stromal-related gene-expression signatures rather than direct measurements of cell abundance, cellular origin, spatial localization, or immune-cell functional state.

### 2.8 Immune checkpoint-related gene analysis

A curated immune-regulatory gene panel (15) was examined to determine whether its expression profile differed between samples classified into CNIH4-high and CNIH4-low groups using the cohort-specific median CNIH4 expression value. Expression distributions were visualized for the two CNIH4 groups and statistically contrasted using a Wilcoxon rank-sum test. These analyses were intended to characterize bulk tumor-level immune-regulatory transcriptional features and were not used to infer the cellular source or spatial organization of individual immune-regulatory signals.

### 2.9 Exploratory pharmacogenomic association analysis

Pharmacogenomic data were obtained from the NCI-60 resource (16). Drug-specific analyses retained cell lines with sufficient response information, whereas compounds with more than 60% missing measurements were not considered. For each eligible compound, Pearson’s correlation coefficient was calculated between CNIH4 expression and the reported IC50 values across the NCI-60 cell-line panel. A complementary median split of CNIH4 expression was used to descriptively compare IC50 distributions between expression strata. Because the NCI-60 panel includes cell lines derived from multiple cancer types and does not represent an HNSCC-specific experimental system, these analyses were considered exploratory cross-cancer pharmacogenomic associations. Lower IC50 values were not interpreted as evidence of CNIH4-dependent drug sensitivity in HNSCC.

### 2.10 Cell culture

SAS and SCC-9 human oral squamous cell carcinoma cell lines were obtained from the Cell Bank of the Chinese Academy of Sciences (Shanghai, China). SAS cells were propagated in Dulbecco’s modified Eagle’s medium, whereas SCC-9 cells were maintained in DMEM/F12. Both culture media contained 10% fetal bovine serum and 1% penicillin–streptomycin. Cells were incubated under humidified conditions at 37 °C with 5% CO_2_ and were routinely screened for mycoplasma contamination.

### 2.11 Small interfering RNA transfection

GenePharma (Suzhou, China) synthesized two siRNAs directed against CNIH4, designated si-CNIH4–1 and si-CNIH4-2, together with a nonspecific negative-control siRNA. The oligonucleotide sequences were: si-NC, sense 5′-UUCUCCGAACGUGUCACGUTT-3′ and antisense 5′-ACGUGACACGUUCGGAGAATT-3′; si-CNIH4-1, sense 5′-CUCUCGGUCUACUUCAUAATT-3′ and antisense 5′-UUAUGAAGUAGACCGAGAGTT-3′; and si-CNIH4-2, sense 5′-GCUGAAGUCACACAUGAAATT-3′ and antisense 5′-UUUCAUGUGUGACUUCAGCTT-3′. The canonical CNIH4 transcript was used as the reference sequence for oligonucleotide annotation. Both si-CNIH4–1 and si-CNIH4–2 were designed to target sequence regions shared by the annotated CNIH4 splice variants and were therefore not intended to selectively suppress a single isoform.

Cells were allowed to reach approximately 70% confluence before transfection. Lipofectamine 3000 was then used to introduce the indicated siRNAs into SAS and SCC-9 cells in accordance with the manufacturer’s protocol.

### 2.12 RNA extraction and quantitative real-time PCR

Cellular total RNA was isolated with the SevenFast Total RNA Extraction Kit for Cells (SM130, SevenBio, China), and RNA quantity and purity were determined using a NanoDrop One spectrophotometer.

Reverse transcription was carried out with the TransScript Uni All-in-One First-Strand cDNA Synthesis SuperMix for qPCR with genomic DNA removal (AU341, TransGen Biotech, China). Quantitative PCR was subsequently performed on a QuantStudio 6 Real-Time PCR System using SYBR Green PCR Master Mix (A25742, Thermo Fisher Scientific, USA).

The thermal protocol comprised 2 min at 50 °C and 10 min at 95 °C, followed by 40 amplification cycles of 95 °C for 15 s and 60 °C for 1 min. GAPDH served as the reference gene, and relative CNIH4 abundance was determined by the 2^−ΔΔCt method (17). The CNIH4 primer sequences were 5′-TCAACTTACCTGTTGCCACTTG-3′ (forward) and 5′-TCTGTTGGATCAAACACTCCA-3′ (reverse).

### 2.13 Cell proliferation assay

Following siRNA treatment, 2,000 SAS or SCC-9 cells were seeded into each well of a 96-well plate. Cell viability-related absorbance was measured after 0, 24, 48, and 72 h. Before each measurement, culture medium was exchanged for 90 μL fresh complete medium supplemented with 10 μL CCK-8 reagent. After incubation for 1 h at 37 °C, absorbance at 450 nm was recorded.

### 2.14 Colony formation assay

Transfected cells were reseeded into 12-well plates at 2,000 cells per well and maintained for 7 days to allow colony development. Cultures were then washed with phosphate-buffered saline, exposed to 4% paraformaldehyde for 30 min, and subsequently stained with crystal violet for 30 min. Plates were imaged after washing and drying, and colony numbers were quantified in ImageJ (18).

### 2.15 Wound-healing assay

Wound closure was evaluated using Ibidi culture inserts positioned in 12-well plates. Transfected SAS or SCC-9 cells were introduced into each chamber at 4 × 10^4^ cells per chamber.

Once a confluent monolayer had formed, the inserts were removed to generate a standardized cell-free area. Microscopic images were collected at 0, 6, and 9 h. ImageJ was used to measure the residual wound area at each time point, and wound closure was calculated as: Migration rate (%) = [(A_0_ − A_t_)/A_0_] × 100%, where A_0_ denotes the wound area at baseline and A_t_ denotes the area at the indicated time point.

Because CNIH4 depletion also influenced cell proliferation, the assay was interpreted primarily as a measure of wound-closure capacity rather than as an isolated measurement of migratory behavior.

### 2.16 RNA sequencing and differential expression analysis

Transcriptomic profiling was performed to characterize molecular changes caused by CNIH4 depletion. SAS cells transfected with si-NC or si-CNIH4–2 were analyzed, with five independent biological replicates included for each condition. Library construction and next-generation sequencing were carried out by Personalbio (Shanghai, China).

Gene-level expression profiles from the two groups were compared to identify differentially expressed genes. Isoform-specific differential expression analysis was not performed. Statistical significance was defined as an adjusted P value <0.05 together with an absolute fold change >1.5. Global variation among samples was examined by principal component analysis, while hierarchical clustering and volcano plots were used to summarize group-specific expression patterns and the distribution of significantly altered genes.

### 2.17 Functional enrichment and gene set enrichment analyses

Hallmark collections were retrieved from the Molecular Signatures Database through the msigdbr package (19). Differentially expressed genes were subjected to over-representation analysis with clusterProfiler (20), and enriched terms were summarized according to adjusted P values and the number of contributing genes.

In parallel, gene set enrichment analysis was performed with clusterProfiler to evaluate coordinated Hallmark pathway changes between the si-CNIH4–2 and si-NC transcriptomes. The sign of the normalized enrichment score was used to distinguish the two directions of enrichment. Multiple testing was controlled by the Benjamini–Hochberg procedure, and pathways with an adjusted P value <0.05 were regarded as statistically significant (21).

### 2.18 Statistical analysis

R was used for analyses derived from public cohorts, while GraphPad Prism was used for the cell-based experiments. Nonpaired two-group comparisons of public data generally employed the Wilcoxon rank-sum test, with the signed-rank test reserved for matched observations. When more than two independent categories were present, an overall Kruskal–Wallis test preceded pairwise comparisons.

Experimental data are presented as mean ± SD from at least three independent biological experiments. Three-group experiments involving si-NC and the two CNIH4 siRNAs were evaluated by one-way ANOVA with Dunnett-adjusted comparisons. Experiments involving both treatment group and time were evaluated using two-way ANOVA followed by multiple comparisons. A two-sided unpaired Student’s t-test was used for experiments containing only two independent conditions.

Survival comparisons used log-rank statistics, whereas continuous linear relationships were summarized by Pearson correlation. All hypothesis tests were two-sided, with P<0.05 regarded as statistically significant. Figure annotations denote *P<0.05, **P<0.01, ***P<0.001, and ****P<0.0001.

Genome-wide RNA-sequencing differential-expression and gene-set enrichment analyses were adjusted for multiple testing using the Benjamini–Hochberg procedure. The clinicopathological, mutation, immune-related, and drug-response analyses were conducted as exploratory association analyses addressing distinct prespecified questions; therefore, nominal two-sided P values are reported. These findings were interpreted as hypothesis-generating rather than definitive evidence of clinical, immunological, or therapeutic relevance.

## 3 Results

### 3.1 CNIH4 is upregulated in HNSCC and shows potential diagnostic value

We first surveyed CNIH4 expression across TCGA malignancies. Expression levels differed across multiple tumor types, and HNSCC was among the cancers showing increased CNIH4 expression relative to normal tissue (**Figure 2A**). A pan-cancer paired comparison of tumor and matched adjacent normal specimens produced a broadly consistent pattern while reducing the influence of between-patient variability (**Figure 2B**). HNSC was included as one cancer type in this overview. CNIH4 abundance also varied considerably among cancer types (**Figure 2C**).

**Figure 2:**
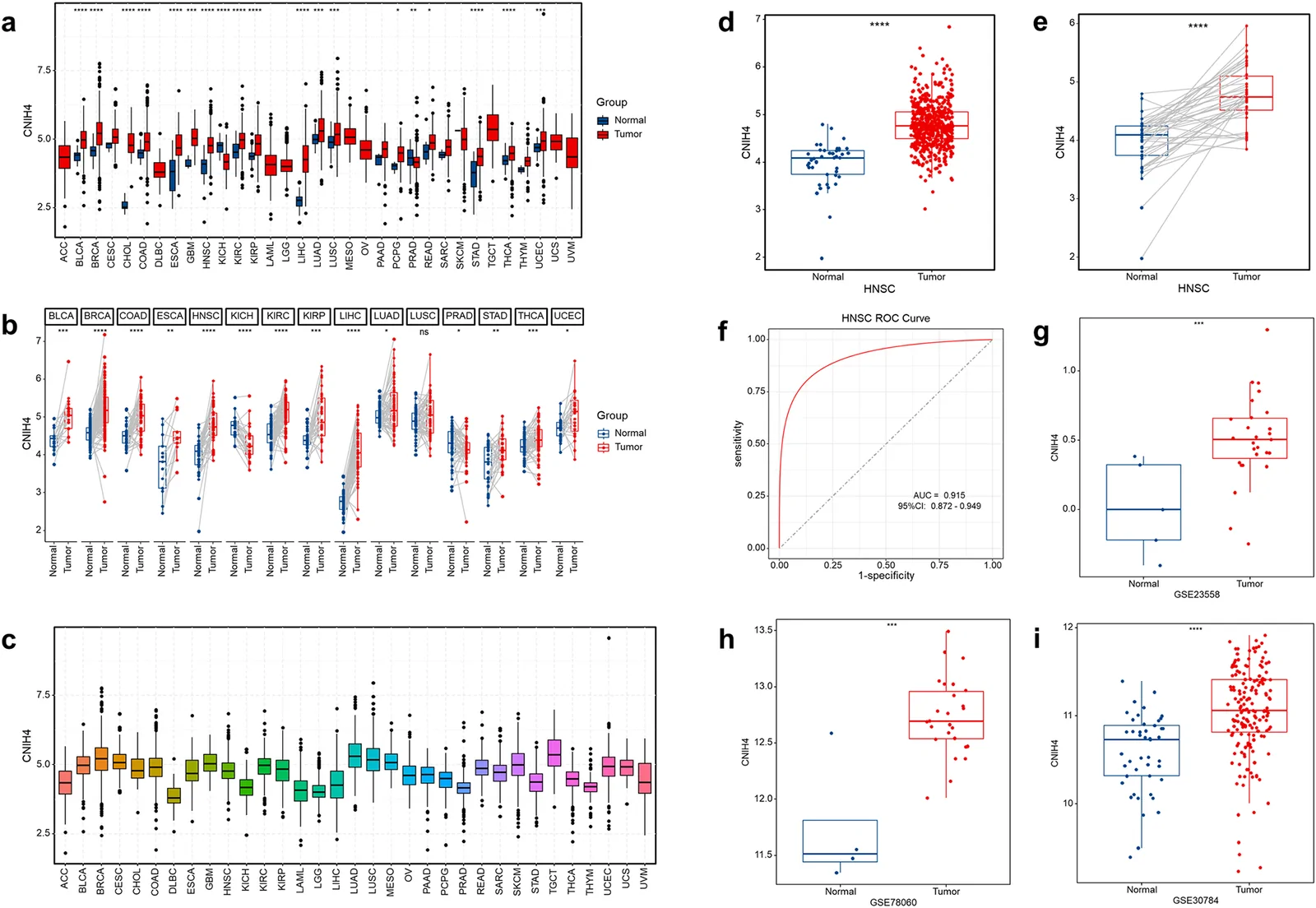
Pan-cancer expression profile, external validation, and diagnostic value of CNIH4 in head and neck squamous cell carcinoma. **(A)** Comparison of CNIH4 expression between tumor and normal tissues across pan-cancer cohorts. **(B)** Pan-cancer paired comparison of CNIH4 expression between tumor tissues and matched adjacent normal tissues across TCGA cancer types with available paired samples. HNSC is included as one cancer type in this overview. Gray lines connect paired samples from the same patient. **(C)** Distribution of CNIH4 expression across different tumor types. **(D)** Comparison of CNIH4 expression between unpaired normal and tumor samples in head and neck squamous cell carcinoma (HNSC). **(E)** Paired comparison of CNIH4 expression between HNSC tumors and matched adjacent normal tissues. **(F)** Receiver operating characteristic (ROC) curve evaluating the ability of CNIH4 expression to distinguish HNSC tumor tissues from normal tissues. The area under the curve (AUC) was 0.915, with a 95% confidence interval of 0.872–0.949. **(G–I)** Validation of differential CNIH4 expression between normal and tumor tissues in the independent GSE23558, GSE78060, and GSE30784 cohorts, respectively. In the box plots, the center line represents the median, the box represents the interquartile range, and individual dots represent individual samples. ns, not significant; *P < 0.05, **P < 0.01, ***P < 0.001, and ****P < 0.0001.

The HNSC-specific paired comparison is shown separately in **Figure 2E** as a focused visualization of the same HNSC result included in the pan-cancer paired analysis in **Figure 2B**. CNIH4 expression was higher in tumor specimens than in matched adjacent normal tissues from the same individuals. ROC analysis yielded an AUC of 0.915 (95% CI, 0.872–0.949), indicating that CNIH4 expression showed good discrimination between malignant and normal tissues in this cohort (**Figure 2F**).

External expression datasets provided further support for this observation. Tumor samples in GSE23558, GSE78060, and GSE30784 consistently exhibited higher CNIH4 expression than their corresponding normal samples (**Figures 2G–I**). Thus, increased CNIH4 expression was reproducible across TCGA-HNSC and three independent GEO cohorts.

### 3.2 CNIH4 expression is associated with selected clinicopathological characteristics

We next examined whether variation in CNIH4 expression was related to clinical characteristics in TCGA-HNSC. No significant expression difference was observed between patients aged ≤60 and >60 years or between female and male patients (**Figures 3A, B**).

**Figure 3:**
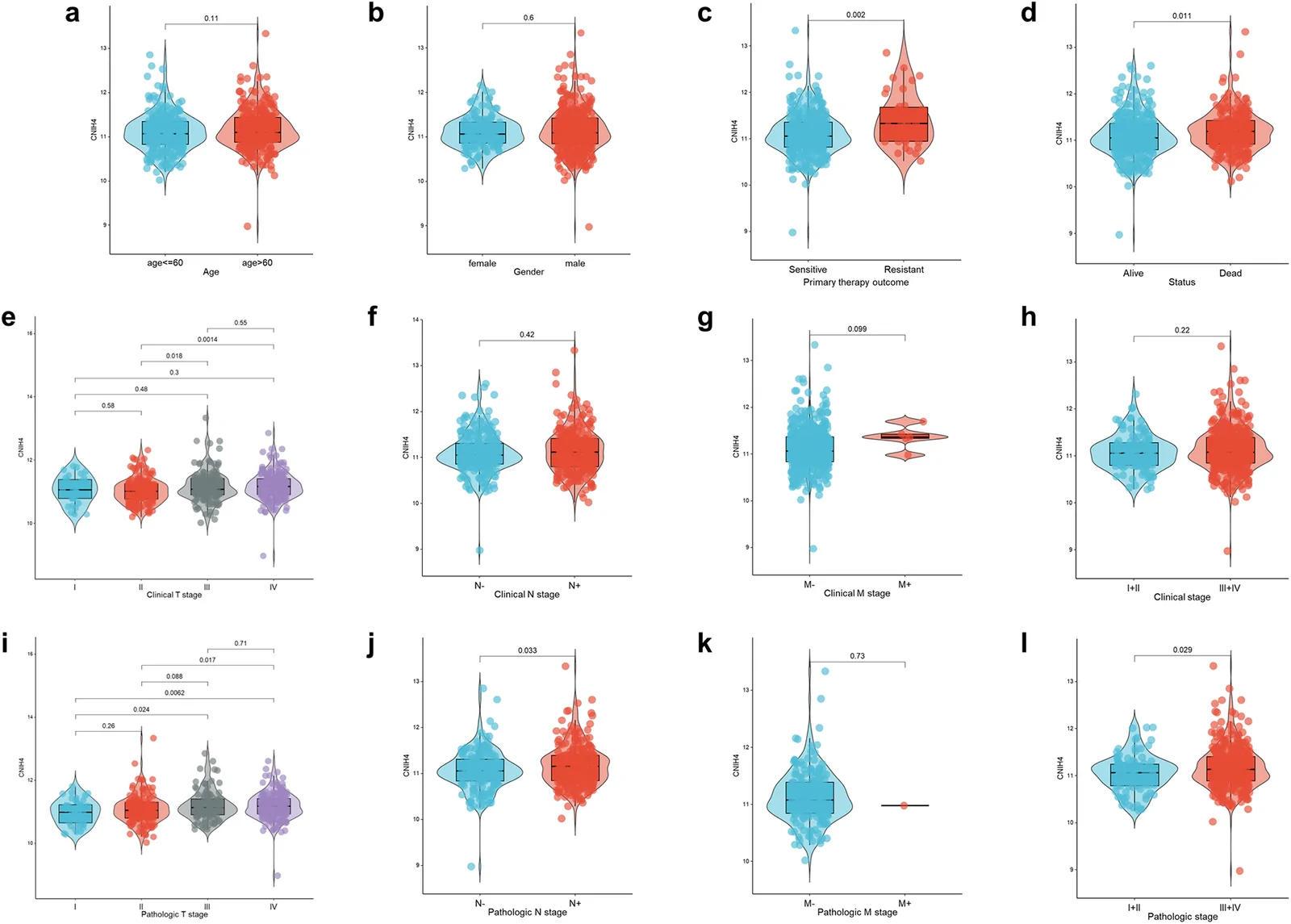
Association between CNIH4 expression and clinicopathological characteristics of patients with head and neck squamous cell carcinoma. CNIH4 expression was compared among different clinicopathological subgroups in the HNSC cohort. **(A)** Age groups (≤60 vs. >60 years); **(B)** sex (female vs. male); **(C)** primary therapy outcome (therapy-sensitive vs. therapy-resistant); **(D)** survival status (alive vs. dead); **(E)** clinical T stage; **(F)** clinical N stage; **(G)** clinical M stage; (h) early versus advanced clinical stage (stages I–II vs. III–IV); **(I)** pathological T stage; **(J)** pathological N stage; **(K)** pathological M stage; and **(L)** early versus advanced pathological stage (stages I–II vs. III–IV). Violin plots show the distribution of CNIH4 expression, the internal box plots indicate the median and interquartile range, and individual dots represent individual patient samples. P values for the corresponding comparisons are shown above the plots.

In contrast, CNIH4 expression was higher among patients classified as resistant to primary therapy than among therapy-sensitive patients (P = 0.002; **Figure 3C**). Patients who had died during follow-up also showed higher CNIH4 levels than those who remained alive (P = 0.011; **Figure 3D**).

Differences were detected among selected clinical T-stage categories, although CNIH4 did not increase progressively with advancing T stage (**Figure 3E**). Clinical N stage, M stage, and grouped clinical stage were not significantly associated with expression (**Figures 3F–H**). A similar nonmonotonic pattern was observed across pathological T categories (**Figure 3I**). By contrast, pathological lymph-node involvement was associated with higher CNIH4 expression (P = 0.033; **Figure 3J**), whereas no significant difference was observed according to pathological M stage (**Figure 3K**). Expression was also elevated in pathological stage III–IV disease relative to stage I–II disease (P = 0.029; **Figure 3L**).

These analyses therefore linked higher CNIH4 expression to therapy resistance, death status, pathological nodal involvement, and advanced pathological stage, while several other conventional staging variables showed no consistent association.

### 3.3 High CNIH4 expression is associated with unfavorable survival outcomes

Pan-cancer univariate Cox analysis was used to determine whether CNIH4 expression was related to overall survival across malignancies. Significant associations were detected in several tumor types (**Figure 4A**). For HNSCC, higher CNIH4 expression corresponded to an increased mortality risk (HR = 1.706, 95% CI 1.241–2.344; P = 0.001).

**Figure 4:**
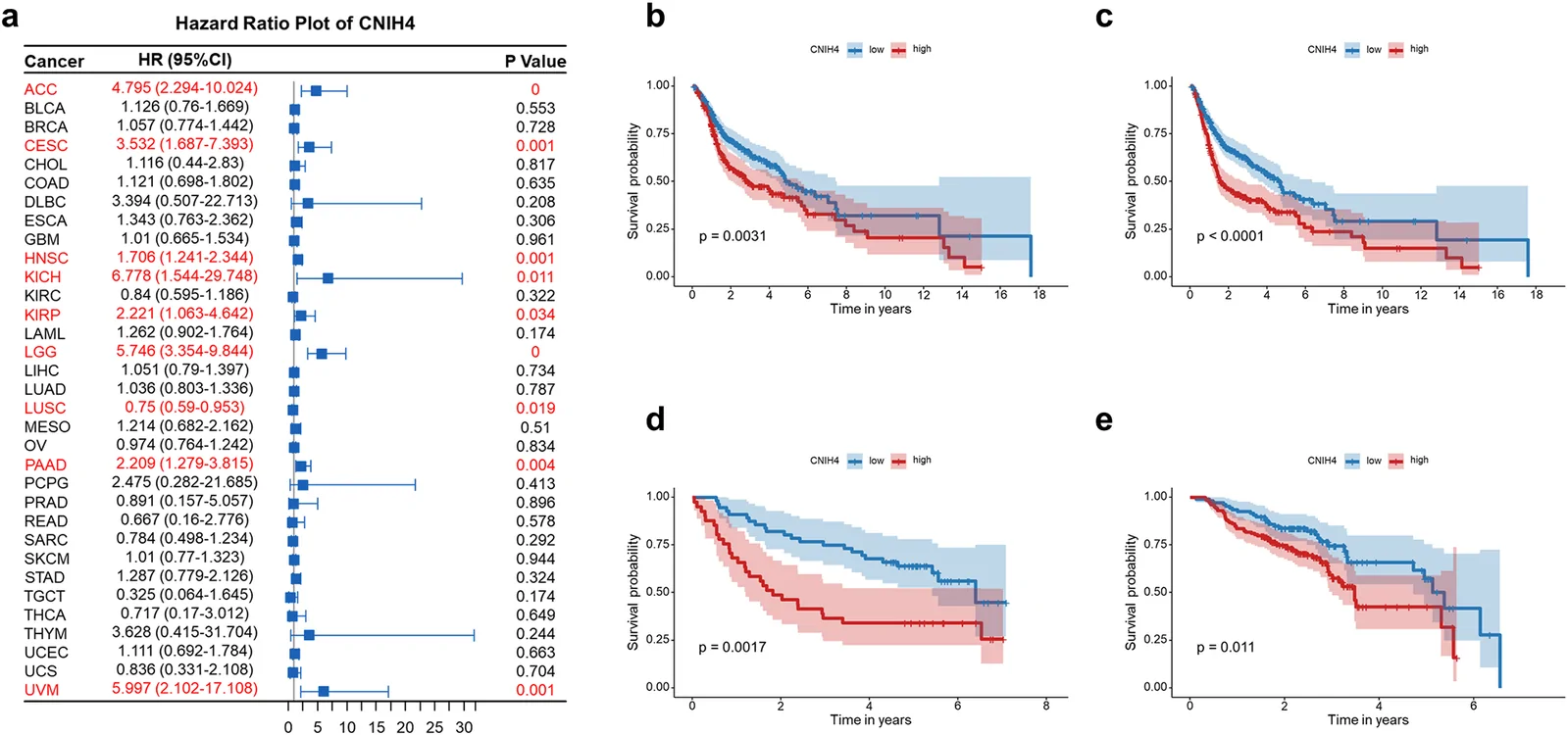
Association of CNIH4 expression with prognosis across cancers and in patients with head and neck squamous cell carcinoma. **(A)** Univariate Cox regression analysis evaluating the association between CNIH4 expression and overall survival across different cancer types. Hazard ratios (HRs) and corresponding 95% confidence intervals (95% CIs) are presented in the forest plot, with statistically significant cancer types highlighted in red. In head and neck squamous cell carcinoma (HNSC), elevated CNIH4 expression was associated with an increased risk of death (HR = 1.706, 95% CI: 1.241–2.344, P = 0.001). **(B–E)** Patients with HNSC were stratified into high- and low-CNIH4-expression groups, and Kaplan–Meier curves were used to compare overall survival (OS, B), disease-specific survival (DSS, C), disease-free interval (DFI, D), and progression-free interval (PFI, E). Shaded areas represent the 95% confidence intervals. Differences between groups were assessed using the log-rank test. High CNIH4 expression was significantly associated with shorter OS, DSS, DFI, and PFI.

The relationship was then examined across multiple HNSCC survival endpoints. Patients in the CNIH4-high group experienced shorter overall survival than those in the CNIH4-low group (P = 0.0031; **Figure 4B**). A pronounced difference was also evident for disease-specific survival (P<0.0001; **Figure 4C**).

Similar patterns were found for disease-free interval (P = 0.0017; **Figure 4D**) and progression-free interval (P = 0.011; **Figure 4E**). Taken together, the four survival analyses consistently associated elevated CNIH4 expression with less favorable clinical outcomes in HNSCC.

### 3.4 CNIH4 expression is associated with distinct somatic mutation patterns and tumor mutation burden

We next explored the genomic context associated with CNIH4. Across cancer types, direct genomic alterations involving CNIH4 were relatively uncommon, although amplification was among the more frequent alteration classes in several malignancies (**Figure 5A**). This pattern indicates that CNIH4 overexpression in HNSCC is unlikely to be attributable solely to alteration of the CNIH4 locus itself.

**Figure 5:**
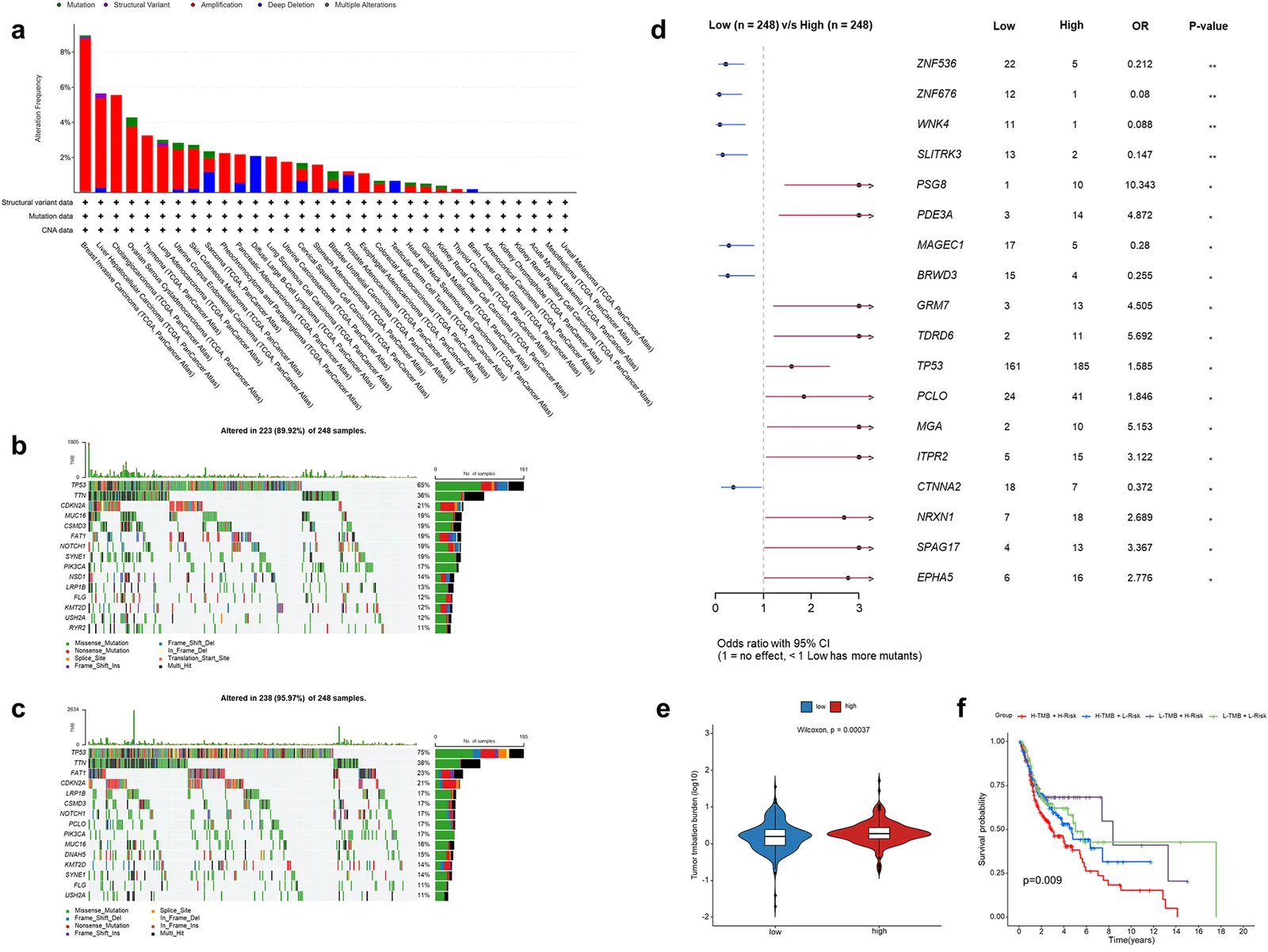
Association of CNIH4 expression with genomic mutation characteristics and tumor mutation burden in head and neck squamous cell carcinoma. **(A)** Frequencies and types of CNIH4 genomic alterations across pan-cancer cohorts, including mutations, structural variants, amplifications, deep deletions, and multiple alterations. **(B, C)** Somatic mutation landscapes of patients with HNSC stratified into the low- and high-CNIH4-expression groups, respectively. The waterfall plots show the most frequently mutated genes and their mutation types, while the bar plots indicate the mutation burden of individual samples and the mutation frequency of each gene. Genomic alterations were detected in 223 of 248 patients (89.92%) in the low-expression group and 238 of 248 patients (95.97%) in the high-expression group. **(D)** Comparison of differentially mutated genes between the low- and high-CNIH4-expression groups. Odds ratios (ORs) and corresponding 95% confidence intervals are shown in the forest plot. An OR < 1 indicates a higher mutation frequency in the low-expression group, whereas an OR > 1 indicates a higher mutation frequency in the high-expression group. The numbers of mutated samples in each group and the corresponding P values are shown on the right. **(E)** Comparison of tumor mutation burden (TMB, log10-transformed) between the low- and high-CNIH4-expression groups. Differences were assessed using the Wilcoxon rank-sum test. **(F)** Kaplan–Meier overall survival curves for patients jointly stratified according to CNIH4 expression and TMB level into high-TMB/high-CNIH4, high-TMB/low-CNIH4, low-TMB/high-CNIH4, and low-TMB/low-CNIH4 groups. Differences among groups were evaluated using the log-rank test. *P < 0.05 and **P < 0.01.

Mutation profiles were then compared after dividing TCGA-HNSC cases by CNIH4 expression. In the low-expression group, 223 of 248 tumors (89.92%) contained at least one somatic mutation, whereas mutations were detected in 238 of 248 tumors (95.97%) in the high-expression group (**Figures 5B, C**). TP53 ranked as the most frequently mutated gene in both groups, with TTN, FAT1, CDKN2A, LRP1B, and CSMD3 also commonly altered.

Several genes differed significantly in mutation frequency between the two expression groups (**Figure 5D**). TP53, PCLO, MGA, ITPR2, NRXN1, SPAG17, and EPHA5 alterations occurred more often in the CNIH4-high group, whereas ZNF536, ZNF676, WNK4, SLITRK3, MAGEC1, and CTNNA2 alterations were enriched in the CNIH4-low group.

TMB was also higher in the CNIH4-high group (Wilcoxon P = 0.00037; **Figure 5E**). When patients were jointly classified according to CNIH4 expression and TMB status, the resulting four subgroups showed significantly different overall survival distributions (P = 0.009; **Figure 5F**). These findings link CNIH4 expression to broader differences in the genomic background of HNSCC.

### 3.5 Construction and evaluation of a CNIH4-based prognostic nomogram

To examine whether CNIH4 could contribute to multivariable survival estimation, we incorporated its expression together with age, clinical T/N/M stage, overall clinical stage, lymphovascular invasion, and perineural invasion into a prognostic nomogram (**Figure 1A**). The total score generated from these variables was used to estimate 1-, 3-, and 5-year overall survival probabilities.

Predicted and observed survival showed generally good agreement on the calibration plots at all three time points (**Figure 1B**). Across most of the follow-up period, the integrated nomogram also showed a higher time-dependent C-index than the risk-score model used for comparison (**Figure 1C**).

Survival differed markedly after patients were classified according to the nomogram score, with the high-score group showing poorer overall survival (P<0.0001; **Figure 1D**). The corresponding time-dependent ROC analysis produced AUC values of 0.710, 0.750, and 0.720 for 1-, 3-, and 5-year overall survival, respectively (**Figure 1E**). Overall, the combined model provided moderate discrimination of HNSCC survival outcomes.

### 3.6 High CNIH4 expression is associated with an altered tumor immune microenvironment

We next examined the tumor microenvironment according to CNIH4 expression. ESTIMATE analysis showed lower composite ESTIMATE scores, immune scores, and stromal scores in the CNIH4-high group than in the CNIH4-low group (P = 0.00022, P = 0.01496, and P = 1.4×10^^−5^, respectively; **Figure 6A**), indicating differences in both immune and stromal content.

**Figure 6:**
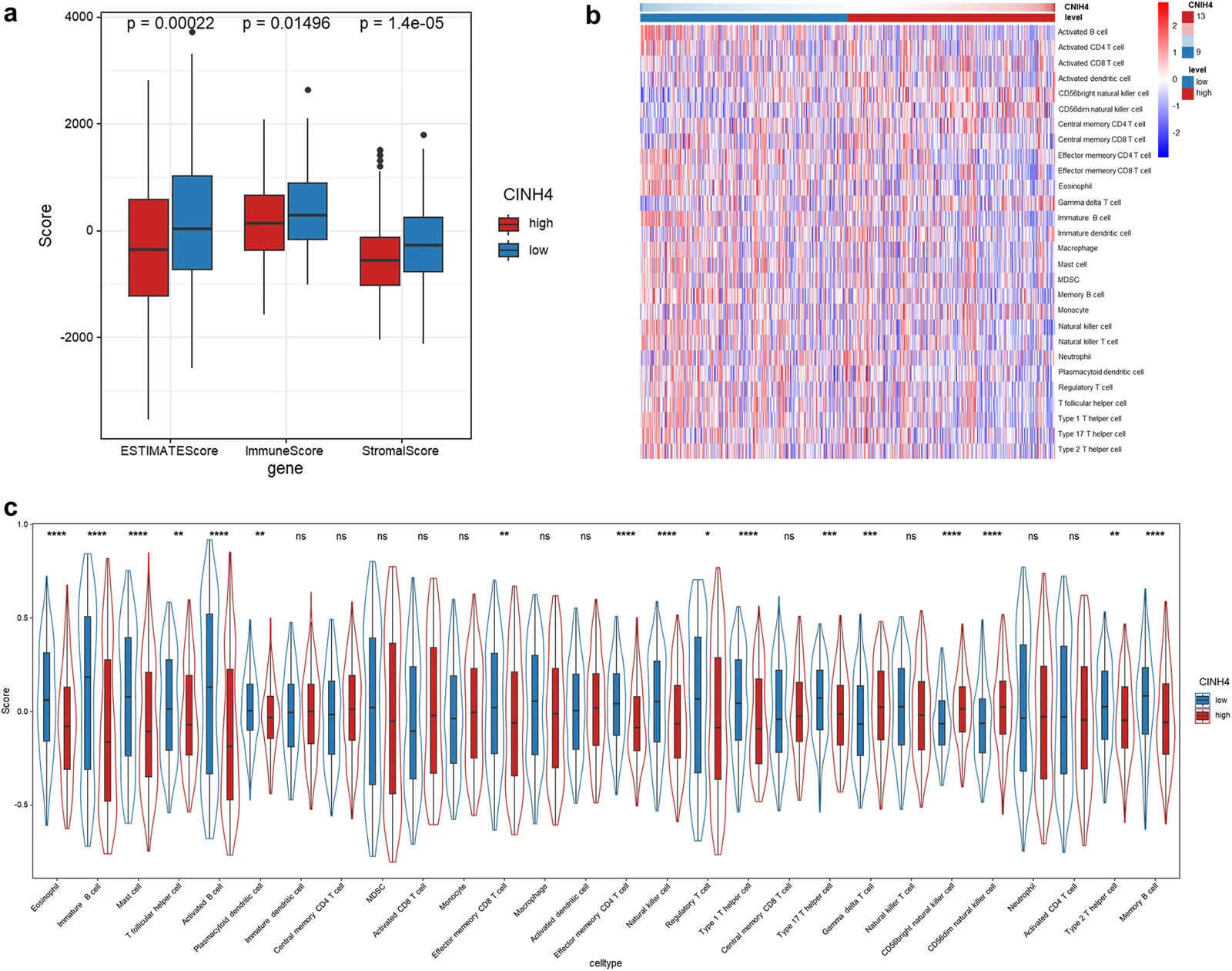
Association of CNIH4 expression with the tumor microenvironment and immune cell infiltration in head and neck squamous cell carcinoma. Patients with HNSC were stratified into high- and low-CNIH4-expression groups. **(A)** Comparison of the ESTIMATE score, immune score, and stromal score between the two groups. The corresponding P values are shown above the plots. **(B)** Heatmap showing the infiltration scores of 28 immune cell types in the high- and low-CNIH4-expression groups. Each column represents an individual patient sample, and each row represents an immune cell type. The annotation bars at the top indicate CNIH4 expression and expression-based grouping. Colors ranging from blue to red represent low to high immune infiltration scores. **(C)** Violin plots comparing the infiltration scores of different immune cell types between the high- and low-CNIH4-expression groups. The violin plots show the score distributions, and the internal box plots indicate the median and interquartile range. Differences between groups were evaluated using the Wilcoxon rank-sum test. Blue indicates the low-CNIH4-expression group, and red indicates the high-CNIH4-expression group. ns, not significant; *P < 0.05, **P < 0.01, ***P < 0.001, and ****P < 0.0001.

Evaluation of 28 immune-cell populations further revealed distinct infiltration profiles between the two groups (**Figure 6B**). Multiple B-cell, T-cell, natural killer-cell, dendritic-cell, and myeloid-cell populations differed significantly according to CNIH4 status (**Figure 6C**), with the high-expression group generally showing lower infiltration scores for several adaptive and innate immune populations.

We then treated CNIH4 expression as a continuous variable rather than dividing tumors into two groups. Most immune-cell populations showed negative correlations with CNIH4, whereas positive correlations were limited to a smaller number of populations, including selected natural killer-cell subsets and γδ T cells (**Figure 7A**).

**Figure 7:**
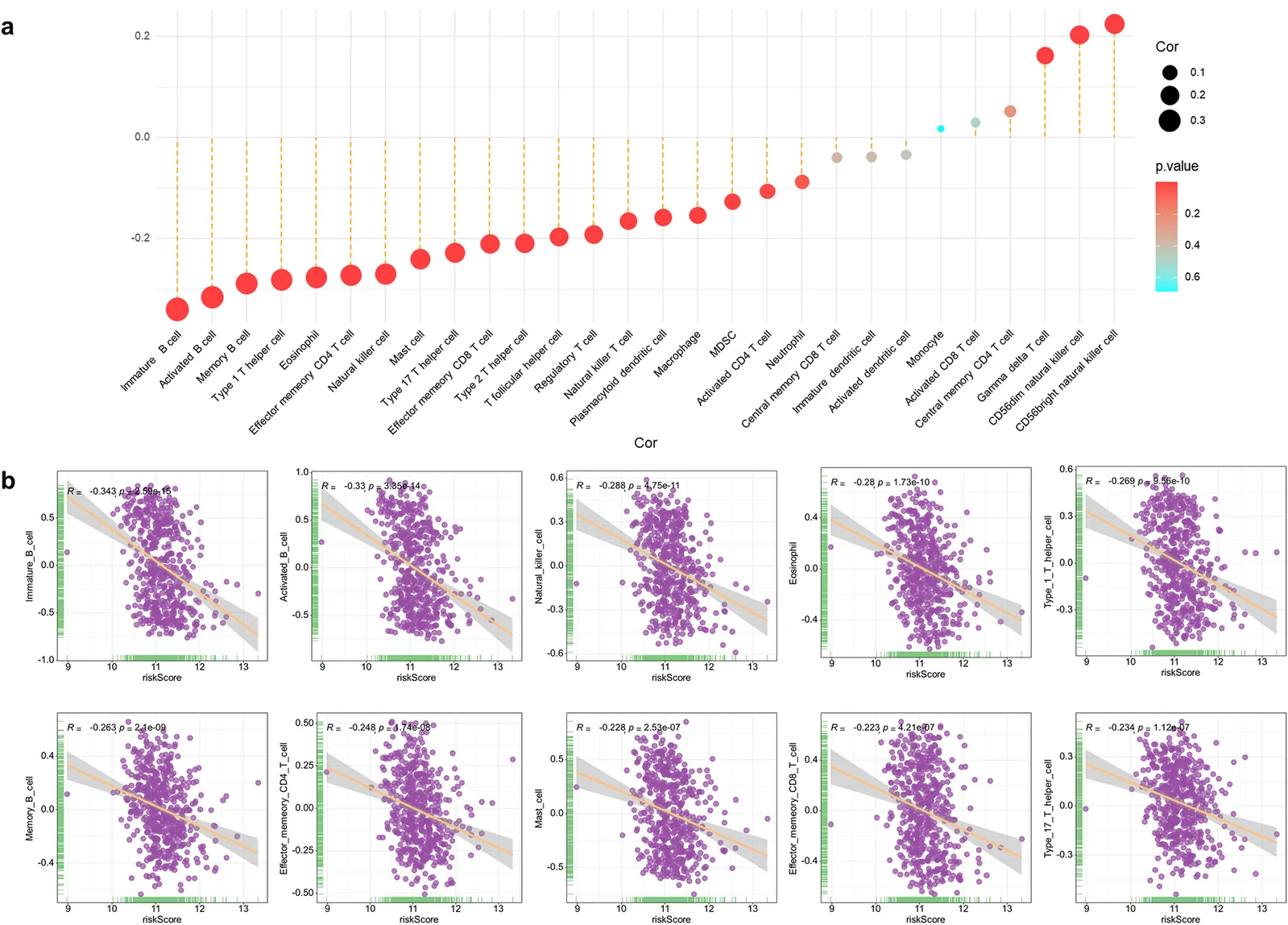
Correlations between CNIH4 expression and immune cell infiltration in head and neck squamous cell carcinoma. **(A)** Correlation analysis between the CNIH4 expression and the infiltration levels of 28 immune cell types. The vertical position of each dot represents the correlation coefficient, dot size indicates the absolute magnitude of the correlation, and dot color represents the corresponding P value. Dashed lines connect the correlation coefficients of individual immune cell types. Positive coefficients indicate that higher CNIH4 expression is associated with higher inferred immune-cell signature scores, whereas negative coefficients indicate an inverse association. **(B)** Scatterplots showing the correlations of the risk score with representative immune cell populations, including immature B cells, activated B cells, natural killer cells, eosinophils, type 1 T helper cells, memory B cells, effector memory CD4^+^ T cells, mast cells, effector memory CD8^+^ T cells, and type 17 T helper cells. Each dot represents an individual patient sample. The orange line indicates the fitted trend, and the gray shaded area represents the 95% confidence interval. Correlation coefficients (R) and corresponding P values are displayed in each panel.

Representative scatterplots illustrated inverse associations with immature B cells, activated B cells, natural killer cells, eosinophils, type 1 T helper cells, memory B cells, effector memory CD4^+ T cells, mast cells, effector memory CD8^+ T cells, and type 17 T helper cells (**Figure 7B**). Overall, the bulk transcriptome-based analyses indicate that higher CNIH4 expression is associated with lower inferred immune and stromal signature scores in TCGA-HNSC. These findings do not establish the cellular source, spatial distribution, or functional state of the corresponding immune and stromal populations.

### 3.7 CNIH4 expression is associated with bulk immune-regulatory transcriptional features and exploratory cross-tumor survival associations in immunotherapy cohorts

To further characterize the immune context associated with CNIH4, we compared a panel of immune-regulatory genes between the two expression groups. Significant differences were observed across multiple major histocompatibility complex genes, costimulatory and coinhibitory molecules, and tumor necrosis factor receptor superfamily members (**Figure 8A**), indicating that CNIH4 status is associated with broader changes in immune-regulatory gene expression.

**Figure 8:**
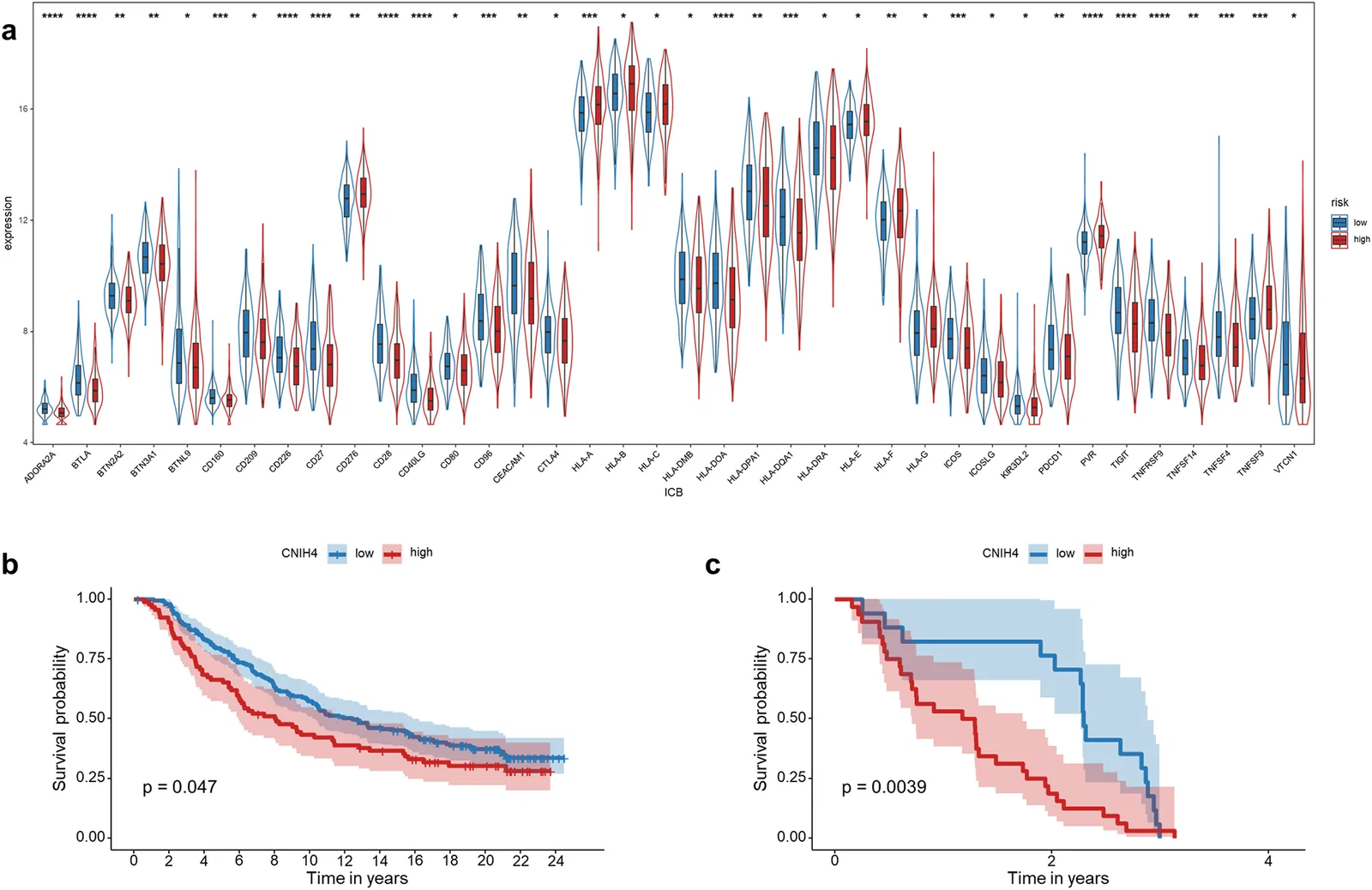
Association of CNIH4 expression with immune checkpoint-related genes and survival outcomes in immunotherapy cohorts. **(A)** Comparison of the expression levels of immune checkpoint-related genes between the high- and low-CNIH4-expression groups. Violin plots show the expression distributions, and the internal box plots indicate the median and interquartile range. Blue represents the low-CNIH4-expression group, whereas red represents the high-CNIH4-expression group. Differences between groups were evaluated using the Wilcoxon rank-sum test. **(B, C)** Kaplan–Meier survival curves for patients stratified into high- and low-CNIH4-expression groups in the IMvigor210 **(B)** and GSE91061 **(C)** immunotherapy cohorts, respectively. Shaded areas represent the 95% confidence intervals, and differences between groups were assessed using the log-rank test. Patients with high CNIH4 expression exhibited lower survival probabilities in these non-HNSCC immunotherapy-treated cohorts. These exploratory survival associations do not establish CNIH4 as a predictive biomarker for immune checkpoint blockade in HNSCC. *P < 0.05, **P < 0.01, ***P < 0.001, and ****P < 0.0001.

We next examined two independent cohorts of patients treated with immune checkpoint blockade. In IMvigor210, higher CNIH4 expression was associated with inferior survival (P = 0.047; **Figure 8B**). The same direction of association was observed in GSE91061, where the high-expression group also had poorer survival (P = 0.0039; **Figure 8C**).

Importantly, neither IMvigor210 nor GSE91061 was derived from HNSCC. Therefore, these results do not validate CNIH4 as an HNSCC-specific predictive biomarker for immune checkpoint blockade and do not establish an association with objective treatment response. Rather, they represent exploratory cross-tumor observations that higher CNIH4 expression was associated with poorer survival in two immunotherapy-treated cohorts. Dedicated HNSCC immunotherapy cohorts with standardized treatment-response and survival data will be required to assess whether CNIH4 has predictive relevance in this disease.

### 3.8 CNIH4 expression shows exploratory associations with drug-response measurements in the NCI-60 panel

NCI-60 drug-response data were used to explore cross-cancer pharmacogenomic associations with CNIH4 expression. Higher CNIH4 expression showed nominal inverse correlations with the reported IC50 values of fluorouracil (Cor=−0.332, P = 0.010; **Figure 9A**), tegafur (Cor=−0.293, P = 0.023; **Figure 9B**), paclitaxel (Cor =−0.264, P = 0.042; **Figure 9C**), and nilotinib (Cor=−0.258, P = 0.046; **Figure 9D**). The median-based expression-group comparisons showed a similar descriptive pattern for these compounds.

**Figure 9:**
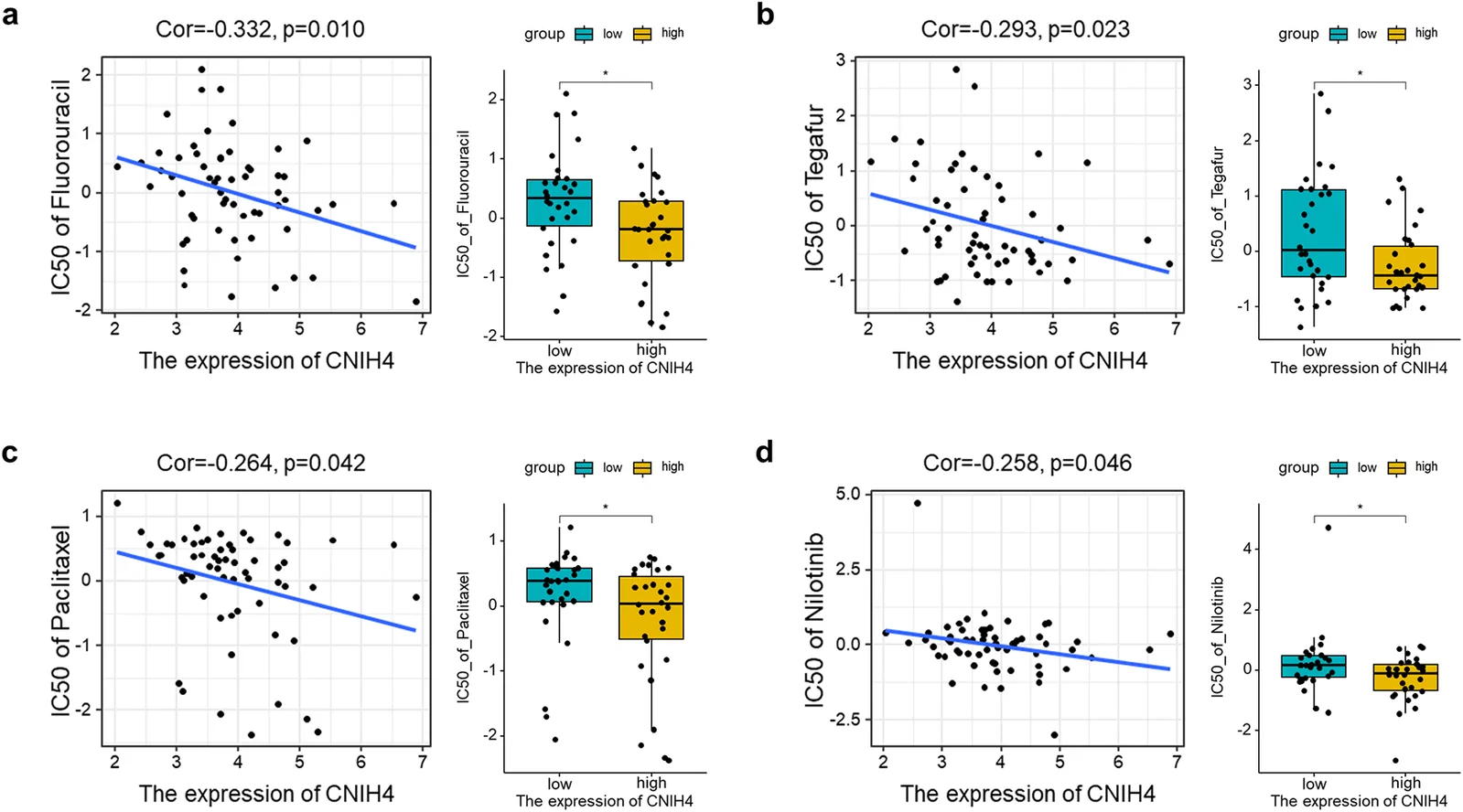
Exploratory associations between CNIH4 expression and drug-response measurements in the NCI-60 cell-line panel. Exploratory pharmacogenomic analyses were performed to assess associations between CNIH4 expression and reported half-maximal inhibitory concentration (IC50) values for selected compounds across the heterogeneous NCI-60 cell-line panel. **(A–D)** Correlations between CNIH4 expression and the predicted IC50 values of fluorouracil **(A)**, tegafur **(B)**, paclitaxel **(C)**, and nilotinib **(D)**, together with comparisons of predicted IC50 values between the high- and low-CNIH4-expression groups. Each dot in the scatterplots represents an individual sample, and the blue line indicates the fitted linear trend. Correlation coefficients and corresponding P values are shown in each panel. In the box plots, the center line represents the median, the box represents the interquartile range, and individual dots represent individual samples. Teal indicates the low-CNIH4-expression group, whereas yellow indicates the high-CNIH4-expression group. CNIH4 expression showed nominal inverse correlations with the reported IC50 values of the displayed compounds. Because these analyses were conducted in a cross-cancer cell-line panel rather than HNSCC-specific models, they should be interpreted as hypothesis-generating associations and not as evidence of CNIH4-dependent drug sensitivity or therapeutic prediction in HNSCC. *P < 0.05.

However, these correlations were modest and were derived from the heterogeneous NCI-60 panel rather than HNSCC-specific models. Therefore, the results do not demonstrate that CNIH4 expression determines drug sensitivity or predicts therapeutic benefit in HNSCC. Instead, they represent hypothesis-generating cross-cancer cell-line associations that require validation in HNSCC models using controlled dose-response experiments after CNIH4 perturbation.

### 3.9 CNIH4 knockdown suppresses the proliferation, colony formation, and migration of oral squamous cell carcinoma cells

We next assessed the effect of CNIH4 depletion experimentally in SAS and SCC-9 cells. Both CNIH4-targeting siRNAs substantially reduced CNIH4 mRNA relative to si-NC in the two cell lines, confirming successful knockdown (**Figure 10A**).

**Figure 10:**
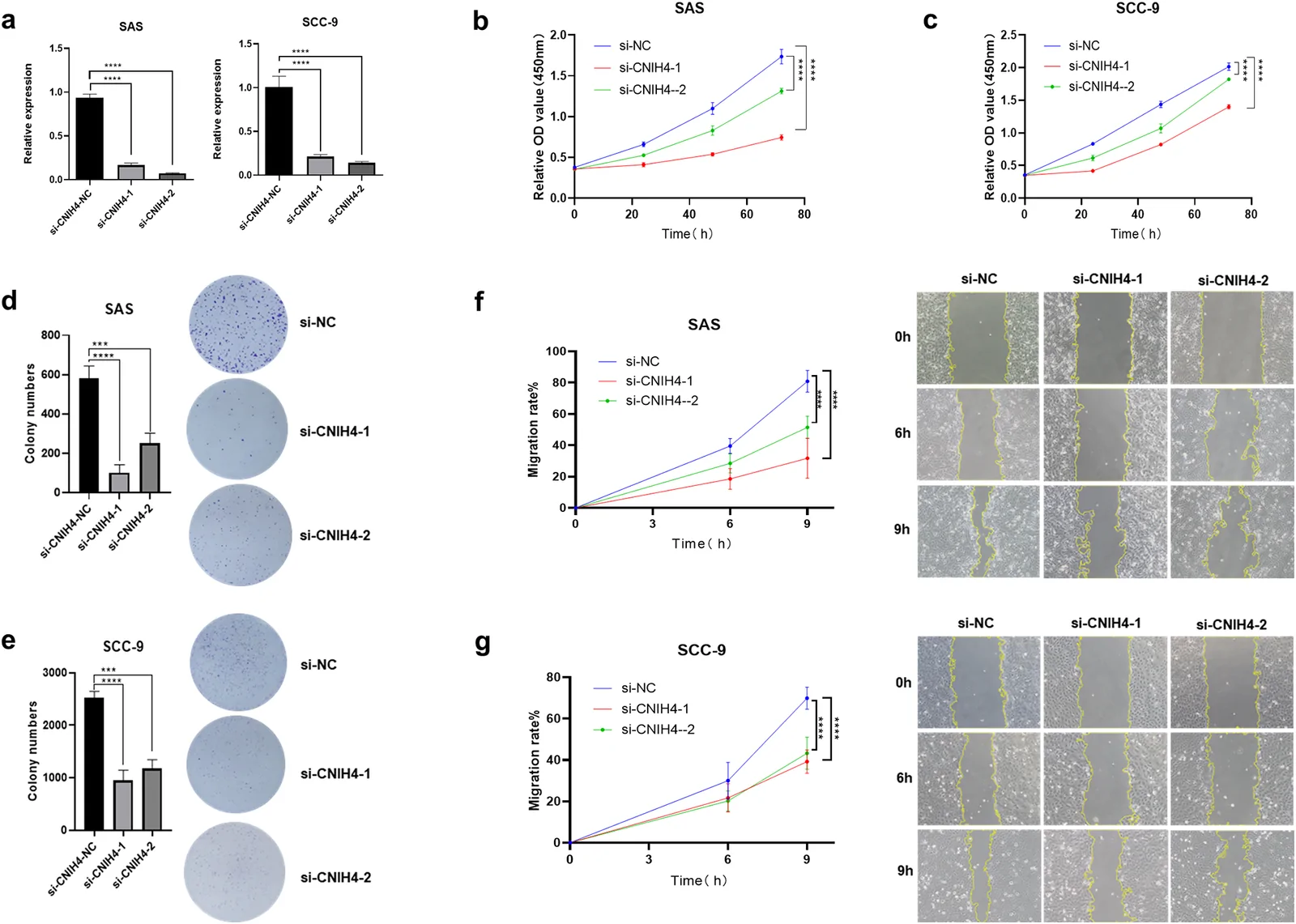
CNIH4 knockdown suppresses the proliferation, colony-forming ability, and migration of oral squamous cell carcinoma cells. **(A)** Relative CNIH4 expression in SAS and SCC-9 cells transfected with negative-control small interfering RNA (si-NC) or two independent CNIH4-targeting small interfering RNAs (si-CNIH4–1 and si-CNIH4-2), as determined by quantitative real-time PCR to assess knockdown efficiency. **(B, C)** CCK-8 assays evaluating the proliferative activity of SAS **(B)** and SCC-9 **(C)** cells at the indicated time points following CNIH4 knockdown. **(D, E)** Representative images and quantitative analyses of colony-formation assays in SAS **(D)** and SCC-9 **(E)** cells after CNIH4 knockdown. **(F, G)** Wound-healing assays evaluating the migration of SAS **(F)** and SCC-9 **(G)** cells following CNIH4 knockdown. Representative images obtained at 0, 6, and 9 h are shown on the right, with the wound margins outlined in yellow, and the quantitative migration rates are shown on the left. Data are presented as the mean ± standard deviation. si-NC, negative-control small interfering RNA; si-CNIH4–1 and si-CNIH4-2, two independent small interfering RNAs targeting CNIH4. *P < 0.05, ***P < 0.001, and ****P < 0.0001.

CCK-8 measurements demonstrated reduced proliferative activity after transfection with both CNIH4-targeting siRNAs in SAS cells (**Figure 10B**), and a comparable pattern was observed in SCC-9 cells (**Figure 10C**). The directionally consistent effects observed with both siRNAs supported the subsequent functional analyses.

Longer-term clonogenic growth was likewise impaired. Both CNIH4-targeting siRNAs reduced colony formation in SAS cells (**Figure 10D**) and SCC-9 cells (**Figure 10E**), indicating that CNIH4 depletion affected not only short-term growth but also the ability of individual cells to establish colonies.

Wound-healing assays provided an additional phenotypic readout. Gap closure proceeded more slowly after CNIH4 knockdown in SAS cells, and quantitative measurements showed reduced wound-closure rates relative to si-NC (**Figure 10F**). The same pattern was reproduced in SCC-9 cells (**Figure 10G**).

Together, these experiments show that reducing CNIH4 expression impairs proliferative activity, clonogenic capacity, and wound closure in two oral squamous cell carcinoma cell lines.

### 3.10 CNIH4-targeting siRNA transfection is associated with transcriptomic changes and candidate cancer-related pathways

RNA sequencing was performed to characterize the molecular consequences of CNIH4-targeting siRNA transfection in SAS cells. Five independent biological replicates were analyzed in each group. The RNA-sequencing dataset additionally provided independent transcriptome-level confirmation of CNIH4 mRNA suppression following si-CNIH4–2 transfection. The normalized CNIH4 counts in the si-NC samples were 1163, 1122, 1097, 998, and 1051, whereas those in the si-CNIH4–2 samples were 321, 266, 266, 267, and 307, respectively (**Supplementary Table S1**). PCA revealed clear separation of the si-NC and CNIH4-knockdown samples, indicating substantial differences in their overall transcriptional profiles (**Figure 11A**). Consistent with the PCA results, hierarchical clustering based on differentially expressed genes distinguished the two experimental conditions (**Figure 11B**).

**Figure 11:**
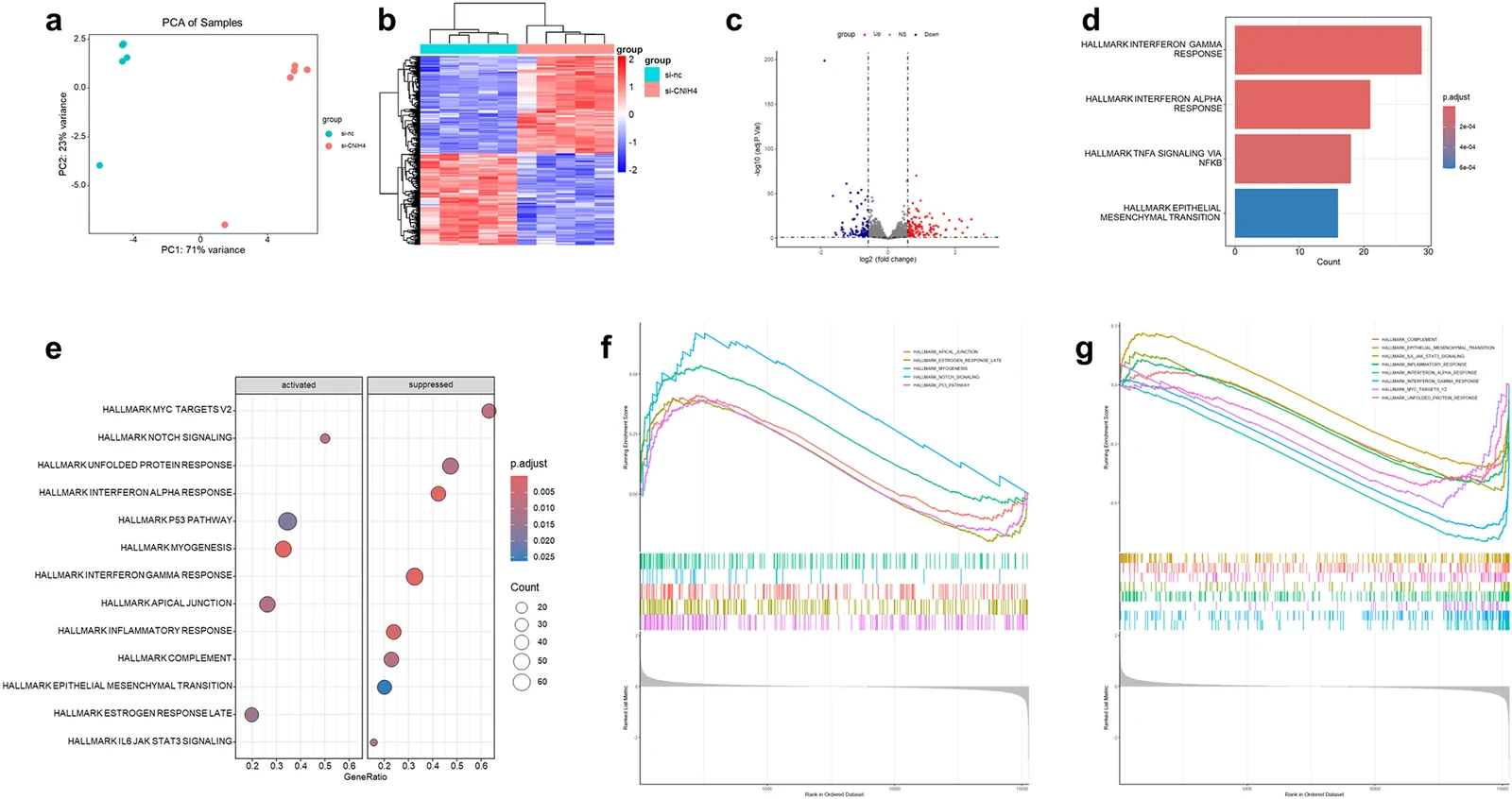
Transcriptomic alterations and pathway enrichment analyses following CNIH4 knockdown. RNA sequencing and bioinformatic analyses were performed in negative-control (si-NC) and CNIH4-knockdown (si-CNIH4) cells. **(A)** Principal component analysis (PCA) showing the overall distribution of transcriptomic profiles in the two groups. Each dot represents an individual sample. **(B)** Hierarchical clustering heatmap of differentially expressed genes. Colors ranging from blue to red indicate low to high scaled expression levels, and the annotation bar at the top indicates the sample groups. **(C)** Volcano plot of differentially expressed genes between the si-CNIH4 and si-NC groups. Red, blue, and gray dots represent upregulated, downregulated, and nonsignificantly altered genes, respectively. **(D)** Hallmark gene-set enrichment analysis of the differentially expressed genes, highlighting interferon-γ response, interferon-α response, TNF-α signaling via NF-κB, and epithelial–mesenchymal transition. Bar length indicates the number of enriched genes, and color represents the adjusted P value. **(E)** Overview of significantly activated and suppressed Hallmark pathways following CNIH4 knockdown. Dot size represents the number of enriched genes, color indicates the adjusted P value, and the x-axis shows the gene ratio. **(F)** Representative positively enriched pathways following CNIH4 knockdown, including apical junction, estrogen response late, myogenesis, NOTCH signaling, and the P53 pathway. **(G)** Representative negatively enriched pathways following CNIH4 knockdown, including complement, epithelial–mesenchymal transition, IL6–JAK–STAT3 signaling, inflammatory response, interferon-α response, interferon-γ response, MYC targets V2, and the unfolded protein response. The pathway analyses identify transcriptomic signatures associated with si-CNIH4–2 treatment and do not establish direct regulation of these pathways by CNIH4.

Numerous genes showed significant expression changes after CNIH4 knockdown, as illustrated by the volcano plot (**Figure 11C**). Hallmark over-representation analysis of the DEG set highlighted interferon-γ response, interferon-α response, TNF-α signaling via NF-κB, and epithelial–mesenchymal transition among the prominent biological programs (**Figure 11D**).

GSEA provided additional information on pathway directionality. Pathways with positive enrichment scores included NOTCH signaling, myogenesis, apical junction, late estrogen response, and the p53 pathway (**Figures 11E, F**). Negative enrichment scores were observed for MYC targets V2, the unfolded protein response, interferon-α response, interferon-γ response, inflammatory response, complement, IL6–JAK–STAT3 signaling, and epithelial–mesenchymal transition (**Figures 11E, G**).

The reduced enrichment of MYC-associated and epithelial–mesenchymal transition signatures was directionally consistent with the reduced proliferative activity and wound-closure capacity observed after CNIH4-targeting siRNA transfection. Similarly, the positive enrichment of the p53 pathway represented a candidate transcriptional change that may be relevant to the growth-associated phenotype. However, these concordant observations do not establish that any individual pathway mediates the phenotypic effects.

Overall, CNIH4-targeting siRNA transfection was associated with broad transcriptomic changes involving proliferation-related, inflammatory, stress-response, cell-junction, and epithelial–mesenchymal transition signatures. These pathway-level findings should be regarded as hypothesis-generating candidate downstream programs. Because rescue experiments and pathway-specific perturbation studies were not performed, the present data do not establish direct regulation of MYC, p53, epithelial–mesenchymal transition, inflammatory signaling, or the unfolded protein response by CNIH4.

## 4 Discussion

The present study extends previous predominantly bioinformatic observations of CNIH4 in HNSCC by integrating public transcriptomic analyses with functional experiments and RNA sequencing. The expression and clinical associations observed in public cohorts should be interpreted as confirmatory or exploratory population-level findings. The principal experimental contribution of this study is that transfection with two independent CNIH4-targeting siRNAs, under conditions of confirmed CNIH4 mRNA suppression, was associated with reduced proliferative activity, clonogenic growth, and wound-closure capacity in two oral squamous cell carcinoma cell lines. RNA sequencing further identified candidate transcriptional programs associated with these phenotypic changes. However, the present data do not establish protein-level target suppression, direct molecular mechanisms, or clinical applicability of CNIH4 as a biomarker.

Increased CNIH4 expression was observed consistently in TCGA-HNSC and in GSE23558, GSE78060, and GSE30784. This agreement across independently generated datasets reduces the likelihood that the expression difference reflects a single cohort or analytical platform. The ROC analysis further indicated good separation of tumor and normal tissues based on CNIH4 expression. However, this AUC was derived from retrospective tissue transcriptomic data and reflects tumor-versus-normal discrimination within the analyzed datasets rather than diagnostic performance in a clinically relevant testing setting. The potential utility of CNIH4 for clinical detection would require validation in prospectively collected specimens and in clinically accessible materials, such as saliva or blood. A previous database-based investigation similarly reported CNIH4 upregulation and prognostic associations in HNSCC (5). Our results therefore confirm this basic expression pattern while extending the analysis through external expression cohorts and experimental validation.

The clinical associations observed in TCGA-HNSC also suggest that CNIH4 expression may reflect biologically aggressive disease. Higher expression was detected in tumors from patients with primary therapy resistance, pathological lymph-node involvement, advanced pathological stage, and death events. Survival analyses were concordant with these findings: elevated CNIH4 was associated with poorer OS, DSS, DFI, and PFI, and pan-cancer Cox analysis also identified an adverse association with OS in HNSCC. Although these results do not establish CNIH4 as an independent or clinically validated prognostic factor, the consistency across multiple outcome measures suggests that CNIH4 expression may represent a candidate molecular feature associated with unfavorable disease behavior. Independent HNSCC cohorts and multivariable validation will be needed before any prognostic application can be considered.

We additionally examined whether CNIH4 could contribute to individualized outcome estimation when considered together with conventional clinical variables. The resulting nomogram showed acceptable calibration and moderate discrimination for 1-, 3-, and 5-year overall survival. Patients assigned higher nomogram scores also experienced substantially poorer survival. These findings suggest that combining molecular and clinicopathological information may offer greater prognostic resolution than considering CNIH4 expression in isolation. However, the model was derived and assessed largely within TCGA-HNSC, and its performance has not yet been established in an independent cohort with complete clinical annotation. The nomogram should therefore be regarded as an exploratory, internally assessed model rather than a tool ready for clinical application. Independent external validation, assessment of calibration and discrimination in separate cohorts, and comparison with established clinicopathological models will be required before clinical use can be considered.

CNIH4 expression was also associated with differences in the genomic landscape of HNSCC. Although direct alterations of the CNIH4 locus were uncommon across cancers, high- and low-expression tumors differed in the frequencies of several recurrently mutated genes. The high-CNIH4 group additionally exhibited greater TMB, and combined stratification using CNIH4 expression and TMB separated patients into groups with different overall survival. TMB has been investigated extensively as a marker of tumor immunogenicity and response to immune checkpoint inhibition (22) but its predictive performance is strongly dependent on tumor context and is not universally reliable (23). Consequently, the association between CNIH4 and TMB in our data is best interpreted as evidence that CNIH4 expression accompanies a distinct genomic background rather than as proof that CNIH4-high tumors are more responsive to immunotherapy.

The immune analyses provide another dimension to this biological context. HNSCC contains a highly heterogeneous tumor microenvironment in which lymphoid, myeloid, and stromal compartments can substantially influence tumor progression and treatment response (24, 25). In our cohort, higher CNIH4 expression was accompanied by lower ESTIMATE, immune, and stromal scores and by lower inferred scores for multiple adaptive and innate immune-cell signatures. Continuous correlation analysis yielded a similar overall pattern, with CNIH4 negatively associated with many immune-cell signature scores. These observations suggest that higher CNIH4 expression is associated with lower bulk transcriptome-derived immune and stromal signature scores in TCGA-HNSC.

Importantly, the present immune analyses were based on bulk tumor transcriptomes and computational deconvolution approaches. Therefore, the observed CNIH4-associated immune and stromal signatures cannot determine whether the relevant transcripts originate from malignant epithelial cells, infiltrating lymphoid or myeloid populations, cancer-associated fibroblasts, or other stromal compartments. Nor can these analyses resolve the spatial distribution, cell–cell interactions, or functional state of the inferred populations. Recent spatial and pathological studies have emphasized the marked compartmental heterogeneity of the HNSCC and OSCC microenvironment. Noda et al. showed that PD-L1/CD274 expression differs across non-tumorous, intraepithelial neoplastic, and invasive HNSCC compartments and further demonstrated the spatially heterogeneous distribution of B7-H4/VTCN1-related signals (26, 27). Li et al. further identified a cancer-associated fibroblast subset associated with restricted CD8+ T-cell infiltration and impaired antitumor activity in HNSCC (28). In OSCC, Liu et al. reported spatially organized metabolic and stromal programs involving inflammatory cancer-associated fibroblast transformation and immunosuppressive interactions, including epithelial–iCAF–Treg crosstalk (29). In this context, our findings do not establish that CNIH4 directly drives immune-cell exclusion, CD8-positive T-cell dysfunction, regulatory T-cell recruitment, or fibroblast-mediated immunosuppression. Rather, CNIH4 expression may be associated with a bulk tumor transcriptional phenotype characterized by lower inferred immune and stromal signatures. Validation using HNSCC tissues with immunohistochemistry, multiplex imaging, single-cell sequencing, and spatial transcriptomics will be required to identify the relevant cellular compartments and clarify whether CNIH4 is directly involved in microenvironmental regulation or is primarily associated with this bulk transcriptional phenotype.

Differences in bulk immune-regulatory gene expression further indicated that CNIH4 status was associated with variation in tumor-level immune-related transcriptional features. However, because bulk expression data cannot identify whether these transcripts originate from malignant cells, infiltrating immune cells, stromal cells, or mixed tissue compartments, the biological basis of these associations remains uncertain. Immune-checkpoint expression alone also does not provide a complete measure of clinical benefit from checkpoint blockade (30, 31). We therefore examined two independent immunotherapy-treated cohorts, in which higher CNIH4 expression was associated with poorer survival. However, IMvigor210 and GSE91061 were derived from non-HNSCC malignancies and were not designed to validate an HNSCC-specific CNIH4 biomarker. Moreover, survival differences in these cohorts should not be interpreted as direct evidence of differential response to immune checkpoint blockade. The present data support only an exploratory cross-tumor association between CNIH4 expression and survival in immunotherapy-treated populations. Dedicated HNSCC cohorts containing pretreatment tissue, treatment-response, and survival data, ideally integrated with histological, single-cell, or spatial characterization, will be needed to determine whether CNIH4 has any predictive relevance for immunotherapy in HNSCC.

Exploratory analysis of the NCI-60 pharmacogenomic resource identified nominal inverse correlations between CNIH4 expression and the reported IC50 values of fluorouracil, tegafur, paclitaxel, and nilotinib. Fluorouracil- and taxane-containing regimens have clinical relevance in selected head and neck cancer settings (32–34); however, this clinical context should not be used to overinterpret the present findings. The NCI-60 panel contains cell lines from diverse tumor types, and the observed correlations were modest. Moreover, these analyses did not involve experimental perturbation of CNIH4 or direct drug-response assessment in HNSCC cells. Therefore, the data do not demonstrate that CNIH4 determines sensitivity to these agents or predicts therapeutic benefit in HNSCC. They should be regarded only as hypothesis-generating cross-cancer pharmacogenomic observations. Controlled dose-response experiments in CNIH4-knockdown and control HNSCC models, followed by validation in clinically annotated cohorts, will be necessary before any therapeutic relevance can be considered.

The cell-based experiments provide functional evidence consistent with a role of CNIH4 in malignant phenotypes of oral squamous cell carcinoma cells. Transfection with two independent CNIH4-targeting siRNAs was associated with reduced proliferative activity, clonogenic growth, and wound-closure capacity in both SAS and SCC-9 cells under conditions of confirmed CNIH4 mRNA suppression. Because cell proliferation can influence wound-healing measurements, the wound-healing results should not be interpreted as a direct and isolated measurement of cellular migration. Rather, they indicate reduced wound-closure capacity under the experimental conditions. The concordant direction of the phenotypic effects observed with two independent siRNAs in two cell lines reduces, but does not eliminate, the possibility of siRNA-specific off-target effects. In addition, the experiments were restricted to oral squamous cell carcinoma models; validation in HNSCC cell lines derived from non-oral anatomical subsites would help define the generalizability of these observations.

CNIH4 has been reported to participate in intracellular trafficking and endoplasmic reticulum export of newly synthesized membrane proteins, including selected G-protein-coupled receptors (3). This function provides a possible biological context for the transcriptional changes observed after CNIH4-targeting siRNA transfection. Altered intracellular trafficking may potentially affect the cell-surface availability of membrane receptors, secretory activity, endoplasmic reticulum homeostasis, and adaptive stress responses. In principle, such alterations could be accompanied by secondary changes in receptor-dependent signaling and transcriptional programs related to proliferation, epithelial plasticity, inflammatory signaling, and proteostasis. This possibility may be relevant to HNSCC because changes in receptor availability and microenvironment-responsive signaling could influence tumor-cell behavior. However, the relevant CNIH4 cargo proteins and GPCR-associated pathways were not identified or directly examined in the present study. Other cornichon family members may also have context-dependent roles in cancer biology. Their cargo specificity, expression patterns, and biological functions, however, may differ across tissues and tumor types. Comparative investigation of CNIH family members in HNSCC may therefore be informative in future studies but was beyond the scope of the present CNIH4-focused work.

RNA-seq analysis identified reduced enrichment of MYC targets, epithelial–mesenchymal transition, inflammatory and interferon-related programs, IL6–JAK–STAT3 signaling, and the unfolded protein response after CNIH4-targeting siRNA transfection, whereas p53 signaling, apical junction, and several other pathways showed positive enrichment. MYC supports proliferation, biosynthetic activity, and metabolic adaptation in cancer cells (35), whereas p53-related programs can constrain cell growth and promote stress responses (36). The direction of these pathway-level changes was broadly compatible with the reduced proliferation and colony formation observed in siRNA-treated cells.

Similarly, lower enrichment of epithelial–mesenchymal transition-related signatures was directionally compatible with reduced wound-closure capacity, as EMT-related programs have been linked to tumor-cell plasticity, motility, invasion, and treatment resistance (37). The altered unfolded protein response signature may also be of interest given the reported role of CNIH4 in intracellular trafficking and endoplasmic reticulum export, as tumor cells often rely on adaptive endoplasmic reticulum stress responses under conditions of high protein-synthesis demand and microenvironmental stress (38). Nevertheless, these observations do not establish that CNIH4 directly regulates GPCR signaling, MYC activity, p53 signaling, epithelial–mesenchymal transition, inflammatory signaling, or the unfolded protein response. We did not identify specific CNIH4 cargo proteins, directly evaluate ER-to-Golgi transport, or test whether altered protein trafficking caused the observed transcriptomic changes. Therefore, the RNA-seq results should be regarded as candidate downstream transcriptional programs associated with CNIH4-targeting siRNA transfection rather than direct CNIH4-regulated mechanisms. Rescue experiments restoring CNIH4 expression and pathway-specific perturbation studies will be required to determine whether any of these signatures causally mediate the observed phenotypes.

Several limitations should be considered when interpreting this study. The clinical, genomic, and immune-related analyses were retrospective and were based largely on publicly available datasets. Several clinicopathological, genomic, immune-related, and pharmacological associations were derived from multiple exploratory comparisons and reported using nominal P values; accordingly, they should be considered hypothesis-generating. In addition, the expression cutoff used for Kaplan–Meier stratification was derived within TCGA-HNSC using outcome information and may have introduced optimism in the apparent survival separation. The prognostic nomogram has not undergone independent external validation. The immune analyses were derived from bulk transcriptomic data and could not resolve the cellular source, absolute abundance, spatial distribution, or functional state of immune and stromal populations. Likewise, the non-HNSCC immunotherapy cohorts and heterogeneous NCI-60 cell-line panel cannot establish immunotherapy prediction or drug sensitivity in HNSCC. Finally, the functional experiments were limited to two oral squamous cell carcinoma cell lines, CNIH4 suppression was confirmed at the mRNA but not protein level, and rescue experiments, pathway-specific perturbation studies, and *in vivo* models were not performed. Future studies integrating protein-level validation, CNIH4 rescue experiments, pathway-focused functional assays, additional HNSCC models, *in vivo* assessment, spatial immune characterization, and clinically annotated independent cohorts will be required to establish the mechanistic and translational significance of CNIH4.

## 5 Conclusion

This study identifies CNIH4 as a molecular feature associated with HNSCC progression and clinical outcome. Increased CNIH4 expression was reproducible across independent datasets and was associated with adverse survival, distinct genomic characteristics, and lower bulk transcriptome-derived immune and stromal signature scores. In oral squamous cell carcinoma models, CNIH4-targeting siRNA transfection under conditions of confirmed mRNA suppression was associated with reduced proliferative activity, clonogenic growth, and wound-closure capacity. Transcriptomic profiling further identified candidate changes in MYC- and p53-related programs, epithelial–mesenchymal transition, inflammatory signaling, and the unfolded protein response; however, direct mechanistic regulation of these pathways by CNIH4 was not established. These findings support further investigation of CNIH4 as a candidate molecular feature associated with malignant phenotypes and clinical outcomes in HNSCC. Its potential therapeutic relevance remains exploratory and will require direct pharmacological validation in HNSCC-specific models, together with *in vivo* and mechanistic studies.

## Data Availability

The public datasets analyzed in this study are available from TCGA and GEO. The RNA-sequencing data generated in this study have been deposited in the NCBI Sequence Read Archive (SRA) under BioProject accession number PRJNA1537533.
